# Discovery-Driven Plasma Proteomics Identifies a Multi-Protein Signature for Amyloid PET Positivity: A Machine Learning Analysis of the Bio-Hermes Cohort

**DOI:** 10.3390/ijms27125533

**Published:** 2026-06-18

**Authors:** Stelios Lamprou, Kalliopi Mavromati, Frank J. Gunn-Moore, Terry J. Quinn

**Affiliations:** 1 School of Cardiovascular and Metabolic Health, University of Glasgow, Glasgow G12 8TA, UK; stelios.lamprou@glasgow.ac.uk (S.L.);; 2 School of Biology, University of St. Andrews, St. Andrews KY16 9AJ, UK; fjg1@st-andrews.ac.uk

**Keywords:** Alzheimer’s disease, amyloid PET, plasma proteomics, machine learning, bioinformatics, disease biomarkers

## Abstract

Alzheimer’s disease is a progressive neurodegenerative disorder in which early detection remains limited by the cost and invasiveness of positron emission tomography and cerebrospinal fluid testing. We evaluated whether plasma proteomic profiles could distinguish amyloid PET-positive from amyloid PET-negative individuals using the Bio-Hermes cohort. After quality control and missing-data filtering, 988 participants and 295 proteins were analysed; 31 proteins showing group differences were used for supervised classification. Random Forest, Gradient Boosting, and Neural Network models were trained across four train/test splits with repeated cross-validation and class downsampling. Amyloid-positive and amyloid-negative groups differed across a subset of proteins, with five remaining significant after false discovery rate correction. Tree-based models performed most consistently, with Random Forest and Gradient Boosting achieving AUC values of 0.79–0.81 and balanced accuracy of 0.68–0.73. Eight proteins (SERPINA1, C3, CRP, APOE4, CFH, VTN, C1QTNF5, and PON1) emerged as recurring high-importance features. These findings indicate that discovery-driven plasma proteomics can identify multi-protein signatures associated with amyloid status and can complement established single-analyte blood biomarkers by adding pathway-level information.

## 1. Introduction

Alzheimer’s disease (AD) is a chronic progressive neurodegenerative disorder that typically begins with memory loss. As the condition progresses, it can affect behaviour, language, spatial awareness, and motor function [1]. It is also the leading cause of dementia [2], with an escalating global burden that requires strategies for earlier and more accurate detection [3].

A central pathological hallmark of AD is the accumulation of amyloid-beta (Aβ) plaques in the brain. Amyloid pathology can be detected using positron emission tomography (PET) or cerebrospinal fluid (CSF) analysis [4,5,6]. These approaches are informative but invasive, expensive, or not widely accessible, limiting their use for routine screening and population-level monitoring [7,8]. This diagnostic gap has increased the interest in peripheral biomarkers, particularly blood-based signatures that reflect central amyloid pathology [9,10].

Plasma proteomics provide a high-throughput and minimally invasive approach to identify systemic biological signatures associated with AD and brain ageing [11,12,13]. When combined with machine learning (ML) and deep learning (DL), proteomic data can be used to discover candidate biomarkers and build predictive models for disease-relevant classification tasks [14,15,16]. Random Forest (RF), Gradient Boosting (GB), and Neural Network (NN) are suitable for high-dimensional biological data because they can model non-linear relationships and interactions among features [17,18].

Supervised ML algorithms learn patterns from labelled data to classify new observations. In predictive modelling, these methods are evaluated using discrimination metrics such as the area under the receiver operating characteristic curve (AUC), together with cross-validation and hyperparameter tuning to reduce overfitting [18,19].

In this study, we analysed plasma proteomic measurements from individuals across the cognitive spectrum, including cognitively unimpaired participants with preclinical amyloid pathology. The primary objective was to assess whether proteomic profiles can distinguish amyloid status, as defined by PET, rather than distinguish clinical AD from cognitively normal status. We aimed to identify proteins associated with amyloid status, use these features in ML-based classifiers, compare feature importance across algorithms, and evaluate the biological relevance of the resulting consensus signature. We hypothesised that a multi-protein plasma signature would distinguish amyloid PET-positive from amyloid PET-negative participants and provide complementary pathway-level information beyond established single-analyte blood biomarkers.

## 2. Results

### 2.1. Patient Characteristics and Demographics

The final analytical cohort included 988 participants after quality control, normalisation, and removal of samples or protein features with missing values. The cohort included 337 amyloid-positive and 651 amyloid-negative individuals (see Table 1).

### 2.2. Sample Overview and Feature Selection

**Figure 1:**
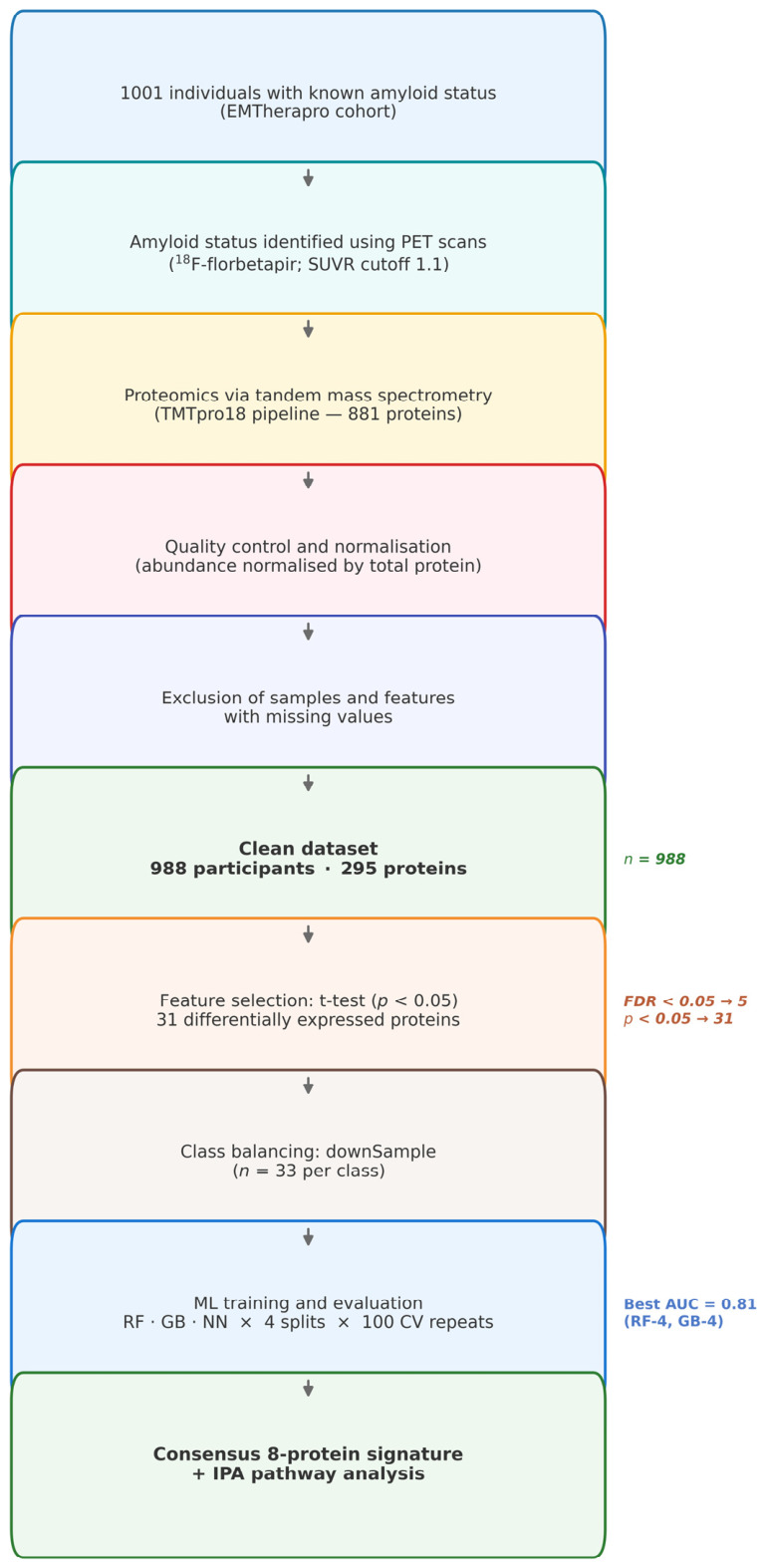
Data preparation, feature selection, and ML pipeline. Plasma proteomics data were filtered for missingness, reduced from 881 measured proteins to 295 complete proteins across 988 participants, screened for amyloid-status group differences, and used to train RF, GB, and NN classifiers across four train/test splits.

Following data curation, as seen in Figure 1, the dataset comprised 988 samples and 295 proteins. Five proteins showed significant differential expression after false discovery rate (FDR) correction: ApoE4 (FDR = 2.299 × 10^−13^), P01024 (FDR = 0.00075117), P08603 (FDR = 0.023618), P02671 (FDR = 0.0340305), and P04004 (FDR = 0.0341628). Because this number was low for predictive modelling, proteins with unadjusted *p*-values < 0.05 were also retained, resulting in 31 proteins for ML training (Table S1).

### 2.3. Machine-Learning Model Performance

**Figure 2:**
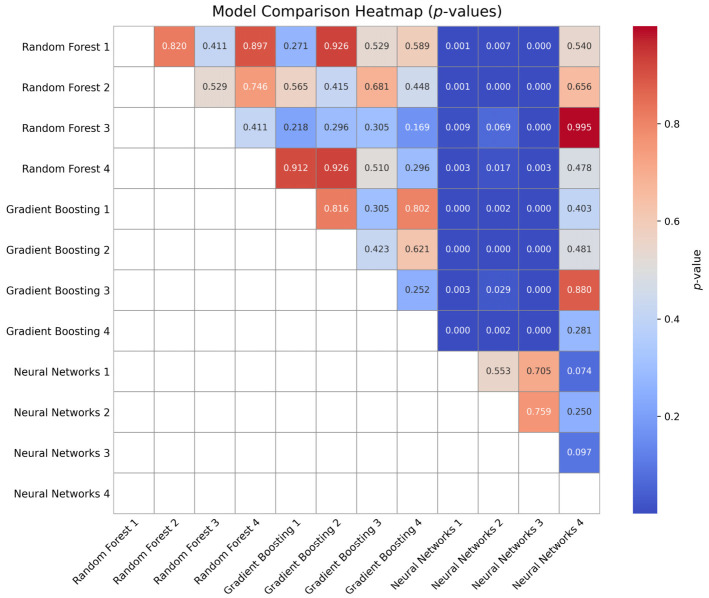
Pairwise comparison of model performance using Benjamini–Hochberg-adjusted DeLong test *p*-values. Pairwise comparisons were performed across the 12 trained classifiers. Lower adjusted *p*-values indicate greater evidence of AUC differences between model pairs.

Model performance is summarised in Table 2. A total of 66 DeLong test comparisons were conducted across the RF, GB, and NN model families. The adjusted *p*-values were visualised as a heatmap (Figure 2), with colour intensity reflecting the degree of statistical significance. RF and GB produced the highest and most stable classification performance across splits. DeLong testing indicated no significant difference between the strongest RF and GB models. AUC values ranged from 0.72 to 0.81 for RF and from 0.73 to 0.79 for GB, with the strongest performance observed in the 80/20 and 90/10 splits. NN models showed lower and more variable performance, with AUC values ranging from 0.53 to 0.71.

### 2.4. Consensus Protein Signature

To identify consistently important proteins, we examined the top 10 ranked features from the best-performing model of each learning method. This threshold was chosen pragmatically to focus on the most influential predictors while limiting noise from lower-ranked variables; it was not derived from the bootstrap analysis, which was used only as a post hoc stability check. Based on overall metrics, the 90/10 split models for RF, GB, and NN (RF-4, GB-4, and NN-4) achieved AUC values ranging from 0.71 to 0.81 and balanced accuracy from 0.66 to 0.73. The top 10 features from these models were extracted and compared (Table S2). As a post hoc descriptive stability check, RF-4 and GB-4 shared eight of their top 10 ranked proteins, corresponding to a Jaccard index of 0.67. Four proteins were shared across the top-10 lists of RF-4, GB-4, and NN-4. In addition, four of the five proteins significant after FDR correction (ApoE4, P01024, P08603, and P04004) were retained in the final eight-protein consensus signature.

To assess whether proteins selected under this top-10 rule were dependent on a single train/test split, we performed a post hoc stratified bootstrap feature-stability analysis using 1000 resamples (Table S3). In each bootstrap iteration, amyloid-positive and amyloid-negative participants were sampled with replacement while preserving class structure, the feature-screening and model-training workflow was repeated, and the top 10 ranked features from RF and GB were recorded. This bootstrap analysis was used to assess stability under the pre-defined top-10 rule, not to choose the threshold itself. ApoE4 was selected among the top 10 features in 100% of both RF and GB iterations. P01024/C3 showed moderate stability, with a combined RF/GB top-10 frequency of 74.0% (95% CI, 72.0–75.8). P02741/CRP and P08603/CFH showed variable but recurrent selection, with combined RF/GB frequencies of 49.0% (95% CI, 46.8–51.2) and 47.5% (95% CI, 45.4–49.7), respectively. The remaining consensus proteins showed lower selection frequencies (P01009/SERPINA1, 34.1%; P04004/VTN, 28.5%; Q13740/PON1, 14.8%; Q6ZS72/C1QTNF5, 9.6%) and should therefore be interpreted as hypothesis-generating. The median RF/GB top-10 Jaccard overlap across bootstrap iterations was 0.54 (IQR, 0.43–0.67), and a median of four final eight-signature proteins were recovered per bootstrap iteration (IQR, 3–5). These findings indicate a stable core discovery signal but variable recovery of the full eight-protein panel.

**Figure 3:**
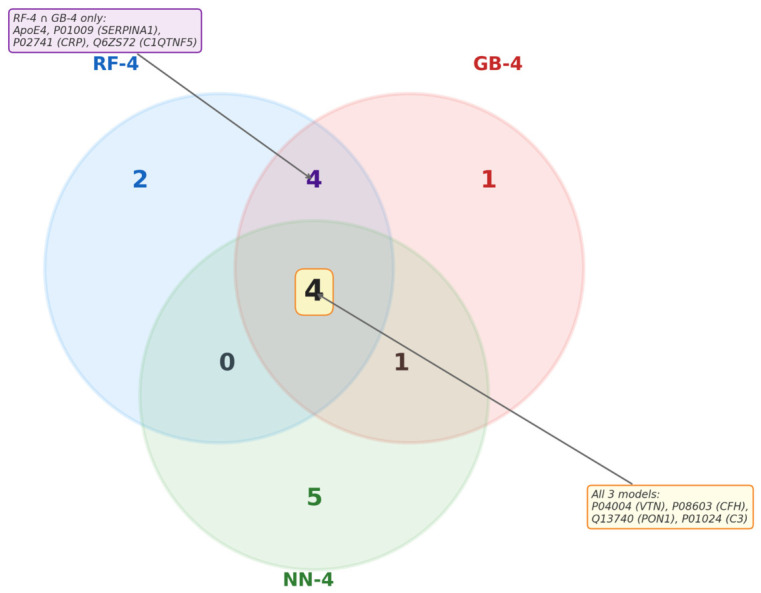
Overlap of Top-10 Proteins Across ML Models. Proteins present across all three classifiers, and proteins shared between the strongest tree-based classifiers, were retained as the consensus proteomic signature.

Four proteins were ranked among the top 10 features across all three classifiers as illustrated in Figure 3: P04004, P08603, Q13740, and P01024. Because RF-4 and GB-4 produced stronger performances than NN-4, their pairwise overlap was also assessed. This added ApoE4, P01009, P02741, and Q6ZS72, yielding an eight-protein consensus signature (Table 3). Four proteins (P01024, P02741, P08603, and Q13740) were upregulated in amyloid-positive individuals, whereas P04004, Q6ZS72, P01009, and ApoE4 were upregulated in the amyloid-negative group.

### 2.5. Protein Association with Dementia and Alzheimer’s Disease

**Figure 4:**
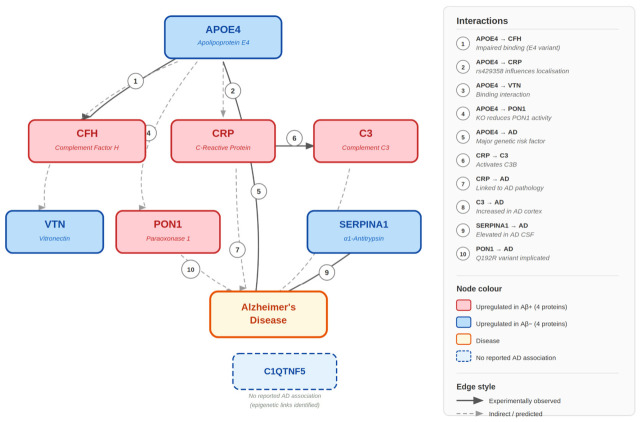
Curated network of the eight consensus proteins and their reported associations with Alzheimer’s disease pathology. Node colour indicates direction of differential expression: red, upregulated in amyloid-positive individuals; blue, upregulated in amyloid-negative individuals. Solid edges represent experimentally observed interactions, whereas dashed edges denote indirect or predicted associations. C1QTNF5 is shown as an isolated node because no direct AD association was identified in IPA, although epigenetic associations have been reported [32].

Ingenuity Pathway Analysis (IPA) outputs of literature-reported associations with AD are illustrated in Figure 4. Upregulation of human SERPINA1 in CSF and increased expression of human C3 in cortical brain tissue have both been associated with AD [20,21]. Elevated plasma CRP, which promotes activation of C3B [22], is linked to AD pathology [23]. APOE4 is strongly associated with AD incidence [24,25], and the APOE rs429358 variant influences the localisation of CRP in serum [26] as well as binding interactions between APOE, complement factor H, and vitronectin [27,28]. IPA did not detect direct AD associations for CFH or VTN in the analysed network.

In mouse models, knockout of Apoe reduces PON1 activity [29], whereas mutation of C3 in the rd10 mouse retina increases Apoe mRNA expression [30]. A mutant form of human PON1 (p.Q192R) has been implicated in AD [31]. No direct association was reported for C1QTNF5 in IPA.

## 3. Discussion

This study shows that plasma proteomic profiling, combined with ML, can classify amyloid status in individuals across the cognitive spectrum, including cognitively unimpaired participants with preclinical amyloid pathology. The models achieved predictive performance using proteomic data alone, without clinical or imaging covariates. The analysis also identified a consensus protein signature with biological links to complement regulation, inflammatory signalling, lipid biology, oxidative stress, and amyloid-related mechanisms.

Among the tested algorithms, decision tree-based models (RF and GB) outperformed NN across most performance metrics. RF-4 and GB-4 were the strongest models, with AUC values of 0.81 and 0.79, respectively. NN showed greater variability and lower predictive reliability. This is consistent with studies in AD and omics-based classification, where ensemble tree models retain stable performance in high-dimensional, smaller-sample settings, whereas neural networks can be more sensitive to sample size and hyperparameter tuning [33,34,35].

The present findings should be interpreted in relation to established blood-based AD biomarkers. Plasma phosphorylated tau 217 (p-tau217) is a leading single-analyte biomarker for amyloid PET prediction, with reported AUC values of 0.85–0.96 across cohorts [36,37,38]. Composite biomarker panels incorporating p-tau217, Aβ42/40, age, and APOE genotype have reported AUC values exceeding 0.95 [39]. These benchmarks exceed the AUC values of 0.79–0.81 observed here. The objectives are different, however. Targeted p-tau217-based approaches are optimised for diagnostic accuracy, whereas the present analysis is discovery-driven and uses high-throughput proteomics to identify multi-protein signatures that may provide pathway-level information.

The consensus protein signature included complement cascade components (C3, CFH), acute-phase reactants (CRP, SERPINA1), oxidative stress mediators (PON1), extracellular matrix-associated proteins (VTN), APOE4, and C1QTNF5. This broader biological profile may complement single-analyte assays by identifying systemic processes associated with amyloid status rather than measuring core amyloid or tau pathology directly.

Several candidate proteins have plausible links to AD mechanisms. CFH and VTN may connect to amyloid and complement regulation indirectly through APOE-related interactions [40,41,42,43]. SERPINA1 has been reported in AD CSF and post-mortem brains in relation to amyloid and tau pathology [40,44]. C3 is increased in AD brain tissue and contributes to synapse loss and neurodegeneration in APP and tauopathy mouse models [45]. CRP has complex associations with cognitive decline and dementia risk, including APOE genotype-specific effects [46,47]. APOE4 remains the strongest genetic risk factor for late-onset AD and influences amyloid clearance, complement regulation, and blood–brain barrier dysfunction [48,49]. PON1 is relevant to oxidative stress, lipid metabolism, and Aβ biology, although genetic association studies have produced mixed findings [50,51,52]. The directionality of the ApoE4 plasma signal should therefore be interpreted cautiously. The measured ApoE4 feature reflects peripheral protein abundance rather than APOE epsilon4 genotype carriage itself, and plasma ApoE abundance may be influenced by lipid transport, inflammatory state, peripheral clearance, and assay-specific isoform detection. Thus, higher ApoE4 abundance in amyloid-negative participants does not contradict the established genetic risk conferred by APOE epsilon4; rather, it indicates that plasma protein abundance and inherited genotype capture related but non-equivalent biological information.

C1QTNF5 is the least characterised candidate in this AD context. No direct peer-reviewed association with AD or amyloid pathology was identified in IPA. However, integrated methylation and gene-expression analyses have reported differential methylation and expression involving C1QTNF5 in AD-related contexts [32]. This supports its status as a hypothesis-generating feature rather than an established AD biomarker.

The main strengths of this analysis are the use of a large, well-characterised plasma proteomics cohort, reproducible preprocessing, fixed random seeds, repeated cross-validation, multiple supervised algorithms, and consensus feature extraction. The approach combines statistical screening with ML classification and pathway-level interpretation, aligning with the discovery aims of proteomics-based biomarker research.

### Limitations and Future Directions

Several limitations are important. First, only five proteins remained significant after FDR correction, and the final ML feature set included proteins selected using unadjusted *p*-values. This increased the available feature space but also increased the risk of Type I error. Second, feature selection was performed before train/test splitting as an exploratory dimensionality-reduction step, rather than within each training fold. This may introduce information leakage because the held-out test data contributed to the initial protein shortlist [53]. Consequently, the reported discrimination metrics should be interpreted as discovery-oriented and potentially optimistic, not as a leakage-free estimate of clinical diagnostic performance. Importantly, the final consensus signature was not based on a single selected model alone: four of the five FDR-significant proteins were retained in the eight-protein consensus signature, and RF-4 and GB-4 shared eight of their top 10 ranked features. The post hoc bootstrap feature-stability analysis further quantified rank stability and supported a stable core centred on ApoE4 and P01024/C3; however, this analysis does not remove potential optimism in the model-performance estimates caused by pre-split feature selection and should not be interpreted as leakage-free validation of model performance. Future validation should embed feature selection within each resampling iteration or use nested cross-validation in an independent cohort. Third, random downsampling discarded a substantial proportion of amyloid-negative samples and may have affected representativeness. Alternative imbalance strategies, including SMOTE, class weighting, or calibration at true prevalence, were not evaluated [54]. Fourth, missing values were handled by listwise deletion, reducing the protein set from 881 to 295 proteins. The complete-case threshold excluded any protein with missing abundance values across retained samples; therefore, 586 of 881 measured proteins (66.5%) were removed. Missingness may reflect technical detection limits or low-abundance proteins rather than random absence, and the possibility that excluded proteins included informative amyloid-related signals cannot be ruled out. Fifth, calibration metrics such as Brier score and calibration plots were not assessed [55]. Sixth, hyperparameter tuning was performed within the same cross-validation framework used for performance estimation rather than through nested cross-validation. Multi-seed model-performance stability analysis was also not performed.

Future iterations should embed feature selection within resampling, evaluate true prevalence performance without downsampling, include calibration assessment, apply nested cross-validation, and compare the discovery-driven proteomic panel directly against established blood biomarkers measured in the same cohort. SHAP-based interpretation could also provide sample-level and direction-aware feature attribution [56,57]. Recent work on AI-based AD classification across cognitively normal and mild cognitive impairment groups, and on metabolic dysfunction in AD biology, further supports the relevance of biologically interpretable predictive modelling across the AD continuum [58,59].

## 4. Materials and Methods

### 4.1. Study Objective and Design

This study assessed whether plasma proteomic profiles can distinguish amyloid-positive from amyloid-negative individuals. The analytical pipeline combined classical statistical testing with supervised ML/DL approaches. The objectives were to identify proteomic features associated with amyloid status and evaluate the predictive performance of classifiers trained on these features.

### 4.2. Data Source

Data were drawn from the Bio-Hermes Study, a multi-centre project designed to build a database of blood-based and digital biomarkers for AD among community-dwelling individuals from diverse backgrounds [60]. The Bio-Hermes Study is registered on ClinicalTrials.gov (NCT04733989). PET imaging with 18F-Florbetapir was used to assess cerebral amyloid burden. Amyloid status was determined using a standardised uptake value ratio (SUVR) cutoff of 1.1, corresponding to a Centiloid value of 24.1. Between April 2021 and November 2022, 1296 participants were enrolled across 17 United States research sites, with 1001 included in the final dataset. Full eligibility and exclusion criteria have been described in the Bio-Hermes primary publication [60].

### 4.3. Data Preparation and Preprocessing

Plasma proteomic measurements were collected from individuals with known amyloid status by EMTherapro (Atlanta, GA, USA), using tandem mass spectrometry with a TMTpro18 pipeline (Thermo Fisher Scientific; Waltham, MA, USA). The provided dataset included 992 participants after quality control and normalisation, with protein abundance normalised by total protein. A total of 881 proteins were initially available in the controlled-access Bio-Hermes proteomics dataset; this participant-level matrix is not provided as Supplementary Material because access is governed by the AD Workbench Data Use Agreement. Protein features and participant samples with any missing abundance values were excluded using a complete-case criterion, yielding 295 proteins and 988 participants for downstream analysis. Thus, 295 of 881 proteins were retained (33.5%) and 586 proteins were excluded (66.5%); 988 of the 992 post-QC participants were retained (99.6%). No additional abundance-dependent imputation threshold was applied. Of these 295 proteins, 31 proteins with amyloid-status group differences were selected for ML training. The final class distribution was 337 amyloid-positive and 651 amyloid-negative participants.

### 4.4. Feature Selection

Two-sample independent t-tests were used to compare protein levels between amyloid-positive and amyloid-negative groups as an exploratory screening step for feature selection. Proteins with FDR < 0.05 were considered significant after multiple-testing correction. Because only five proteins remained significant after correction, proteins with unadjusted *p*-value < 0.05 were also retained to support ML training, resulting in 31 selected proteins. This statistical screening step was performed before model training and train/test partitioning. It was used as exploratory dimensionality reduction for a discovery-oriented proteomic analysis rather than as a locked clinical prediction pipeline.

### 4.5. Machine-Learning Pipeline

RF, GB, and NN classifiers were used to predict amyloid PET status. Each algorithm was trained and tested across four train/test splits: 60/40, 70/30, 80/20, and 90/10. A fixed random seed was used (set.seed(123)). Class imbalance was addressed using the downSample function in the caret package [version 7.0-1], which randomly reduced the majority class to the size of the minority class (*n* = 337).

Hyperparameter tuning and repeated cross-validation were conducted within each algorithm. The caret package in R was used for model training, with tuneLength specifying the number of hyperparameter values assessed. Stability and generalisability were evaluated using 10-fold repeated cross-validation, repeated 100 times. Hyperparameter tuning was performed within the same cross-validation framework used for performance estimation, rather than through nested cross-validation.

Confusion matrices and receiver operating characteristic (ROC) curves were generated for each model. Performance metrics included AUC, sensitivity, specificity, balanced accuracy, PPV, and NPV. DeLong’s test for correlated ROC curves was used to compare AUCs, with Benjamini–Hochberg adjustment for multiple comparisons.

Data preprocessing, manipulation, analysis, and visualisation were performed in R version 4.6.0. Core workflows used caret version 7.0-1, tidyverse version 2.0.0, dplyr version 1.2.1, ggplot2 version 4.0.3, ranger version 0.18.0, gbm version 2.2.3, and nnet version 7.3-20. Receiver operating characteristic curve analysis and DeLong tests were performed using pROC version 1.19.0.1.

### 4.6. Feature Integration and Comparative Analysis

The top 10 proteins from the best-performing RF, GB, and NN models were compared. This threshold was chosen pragmatically because importance scores showed a steep decline after the first 10 variables and because it restricted interpretation to the highest-ranked features while limiting noise from lower-ranked variables. To assess whether proteins selected under this top-10 rule were dependent on a single train/test split, a post hoc stratified bootstrap feature-stability analysis was then performed using 1000 resamples. Bootstrap samples were generated with replacement while preserving amyloid-positive and amyloid-negative class structure. Within each bootstrap iteration, the t-test-based feature-screening step was repeated, RF and GB models were retrained, and the top 10 ranked features from each tree-based classifier were extracted. Feature stability was summarised as the proportion of bootstrap iterations in which each protein appeared among the top 10 features, together with median rank and interquartile range among iterations in which the protein was selected. For top-10 selection frequencies, 95% confidence intervals were calculated using Wilson binomial confidence intervals. For combined RF/GB stability estimates, RF and GB bootstrap iterations were pooled, giving 2000 model-iterations per protein. Agreement between RF and GB top-10 feature sets was summarised using the Jaccard index. In caret, RF variable importance is derived from the mean decrease in Gini impurity or classification accuracy, GB importance is based on improvements in loss function across trees, and NN importance is derived from connection weights between input and hidden layers. Overlapping features were visualised using Venn diagrams. Proteins present in all three top-performing models, or shared between the strongest RF and GB models, were retained as the consensus proteomic signature.

### 4.7. Pathway and Disease Association Analysis

The Pathway Analysis function of Ingenuity Pathway Analysis (IPA; QIAGEN, Manchester, UK) was used to assess biological relevance of shortlisted proteins. Analyses were conducted using default settings, species set to Homo sapiens, with no restriction by tissue category or biological source within the IPA Knowledge Base. The Connect-Pathway function was used to examine molecular interactions, canonical pathways, and disease-associated networks enriched in the protein set. No experimental cell lines or non-model plant species were used in this secondary analysis.

The IPA Pathways and Diseases and Functions modules were used to assess whether shortlisted proteins had documented associations with AD, dementia, or neurodegenerative processes. Networks and annotations were filtered for neurological disease relevance, and high-confidence experimentally observed relationships were prioritised. Enrichment was assessed using Fisher’s exact test, with *p*-values < 0.05 considered significant.

### 4.8. Data, Code, Ethics, and Reproducibility

Bio-Hermes Data Challenge data are publicly available through the Alzheimer’s Disease Data Initiative platform via the AD Discovery Portal on AD Workbench. General code for the ML pipeline is available at GitHub: https://github.com/stelioslamprou37/SML_PipelineR (accessed on 2 September 2025). Model training and testing procedures used fixed seeds and documented preprocessing steps. Reporting followed TRIPOD+AI principles where applicable [61].

This study constitutes a secondary analysis of fully de-identified data from the Bio-Hermes Study [60] and did not involve new participant recruitment, participant contact, intervention, or collection of human samples by the present authors. The original Bio-Hermes Study was conducted in accordance with the Declaration of Helsinki; the protocol was reviewed and approved by Advarra, a central Institutional Review Board (Reference Number Pro00046018), and registered on ClinicalTrials.gov (NCT04733989). Access to the de-identified data was granted under the AD Workbench Data Use Agreement administered by the Alzheimer’s Disease Data Initiative. Parent-study ethics information is reported in the original Bio-Hermes publication, “The Bio-Hermes Study: Biomarker database developed to investigate blood-based and digital biomarkers in community-based, diverse populations clinically screened for Alzheimer’s disease” (PubMed ID: 38415908; accessed on 2 March 2026).

## 5. Conclusions

This work presents a reproducible framework for using plasma proteomic data and ML to predict amyloid status across the cognitive spectrum. A shared proteomic signature across top-performing models supports the value of blood-based proteomics for non-invasive biomarker discovery. The results validate expected markers such as APOE4 and identify less characterised proteins, including C1QTNF5, as hypothesis-generating candidates. Although this discovery-driven approach does not match the diagnostic accuracy of established single-analyte assays such as p-tau217, it provides complementary pathway-level information that may support future biomarker discovery and mechanistic investigation in AD.

## Figures and Tables

**Table 1:** Demographics of the Bio-Hermes/EMTherapro analytical cohort.

Characteristic	Value
Total sample size	988
Age (years)	Range: 59–85; mean: 72; median: 72
Sex, % (*n*)	Female: 56% (553); male: 44% (435)
Race/ethnicity, % (*n*)	White: 85.6% (845); Black or African American: 11.5% (113); Asian: 1.9% (19); American Indian or Alaska Native: 0.2% (2); Native Hawaiian or Other Pacific Islander: 0.1% (1); unknown: 0.8% (8)
Amyloid status, % (*n*)	Positive: 34% (337); negative: 66% (651)

**Table 2:** Machine-learning performance metrics across model families and train/test splits.

Metric	RF1	RF2	RF3	RF4	GB1	GB2	GB3	GB4	NN1	NN2	NN3	NN4
AUC	0.79	0.74	0.72	0.81	0.78	0.76	0.73	0.79	0.53	0.62	0.58	0.71
NPV	0.78	0.73	0.64	0.74	0.77	0.72	0.63	0.71	0.58	0.61	0.57	0.64
PPV	0.63	0.68	0.69	0.72	0.62	0.67	0.64	0.69	0.51	0.54	0.52	0.67
BA	0.71	0.69	0.64	0.73	0.70	0.68	0.62	0.71	0.54	0.58	0.55	0.66
Specificity	0.62	0.67	0.71	0.64	0.61	0.66	0.68	0.68	0.48	0.53	0.34	0.67
Sensitivity	0.81	0.71	0.57	0.82	0.79	0.71	0.57	0.74	0.59	0.63	0.76	0.64
Accuracy	0.69	0.68	0.63	0.71	0.68	0.67	0.61	0.70	0.52	0.57	0.56	0.66

AUC, area under the receiver operating characteristic curve; BA, balanced accuracy; GB, Gradient Boosting; NN, Neural Network; NPV, negative predictive value; PPV, positive predictive value; RF, Random Forest. Performance was evaluated on held-out test sets. Confidence intervals for AUC were not computed, and this is noted as a limitation.

**Table 3:** Proteins with predictive ability for amyloid positivity.

UniProt ID	Protein Name	Gene Symbol	Function
P04004	Vitronectin	VTN	Involved in cell adhesion, coagulation, and inhibition of the membrane attack complex.
P08603	Complement factor H	CFH	Regulates the complement system to prevent damage to host cells.
Q13740	Serum paraoxonase/aryl-esterase 1	PON1	Detoxifies organophosphates and protects lipoproteins from oxidative damage.
P01024	Complement C3	C3	Central component of the complement cascade; marks pathogens for destruction.
P01009	Alpha-1-antitrypsin	SERPINA1	Protease inhibitor that protects tissues from enzymes of inflammatory cells.
P02741	C-reactive protein	CRP	Acute-phase protein that binds to phosphocholine on dying cells and pathogens.
ApoE4	Apolipoprotein E, isoform E4 variant	APOE	Lipid transport and injury repair in the central nervous system; E4 isoform linked to Alzheimer’s risk.
Q6ZS72	Complement C1q tumour necrosis factor-related protein 5	C1QTNF5	Involved in immune response and retinal structure maintenance.

Group 1, amyloid-negative; Group 2, amyloid-positive. A positive t-value indicates upregulation in the amyloid-negative group, whereas a negative t-value indicates upregulation in the amyloid-positive group.

## Data Availability

The Bio-Hermes plasma proteomic dataset and associated phenotypic data are available through the AD Workbench platform of the Alzheimer’s Disease Data Initiative (https://www.alzheimersdata.org/) under a controlled-access Data Use Agreement. Participant-level proteomic data are not provided as Supplementary Material because access is governed by the original data-use terms. The R analysis code supporting the findings of this study is openly available on GitHub at https://github.com/stelioslamprou37/SML_PipelineR (accessed on 16 June 2026).

## Supplementary Materials

The following supporting information can be downloaded at: https://www.mdpi.com/article/10.3390/ijms27125533/s1.

## Abbreviations

The following abbreviations are used in this manuscript:

AD	Alzheimer’s disease
ADDI	Alzheimer’s Disease Data Initiative
APOE4	Apolipoprotein E4
APP	Amyloid precursor protein
AUC	Area under the receiver operating characteristic curve
Aβ	Amyloid-beta
BA	Balanced accuracy
BH	Benjamini–Hochberg
CFH	Complement factor H
CRP	C-reactive protein
CSF	Cerebrospinal fluid
DL	Deep learning
FDR	False discovery rate
GB	Gradient Boosting
GFAP	Glial fibrillary acidic protein
IPA	Ingenuity Pathway Analysis
ML	Machine learning
NfL	Neurofilament light chain
NN	Neural Network
NPV	Negative predictive value
PET	Positron emission tomography
PON1	Paraoxonase 1
PPV	Positive predictive value
RF	Random Forest
ROC	Receiver operating characteristic
RR	Random Repeats
SERPINA1	Serpin family A member 1/alpha-1-antitrypsin
SHAP	SHapley Additive exPlanations
SMOTE	Synthetic Minority Oversampling Technique
SUVR	Standardised uptake value ratio
TRIPOD+AI	Transparent Reporting of a multivariable prediction model for Individual Prognosis Or Diagnosis plus Artificial Intelligence
VTN	Vitronectin
