# Supplementary material for: Discovery-Driven Plasma Proteomics Identifies a Multi-Protein Signature for Amyloid PET Positivity: A Machine Learning Analysis of the Bio-Hermes Cohort
Source: Int J Mol Sci. 2026 Jun 18;27(12):5533. doi: 10.3390/ijms27125533 (PMC13299064; doi:10.3390/ijms27125533)
Supplement: Supplementary file 1 [file ijms-27-05533-s001.zip › ijms-4331143-supplementary.pdf]

Supplementary Materials

Table S1. Differential-expression results for statistically shortlisted plasma proteins by amyloid PET status.

Directionality note: Group 1 = amyloid-negative and Group 2 = amyloid-positive.

Protein	t-value	Degrees of freedom	p-value	FDR
ApoE4	-8.307304	544.3554	0.000000000000000782	0.0000000000002299
P01024	4.597768	668.4067	0.00000511	0.00075117
P08603	3.691254	671.9609	0.000241	0.023618
P02671	-3.527214	454.9060	0.000463	0.0340305
P04004	3.456623	671.6612	0.000581	0.0341628
P01042	3.099841	671.7806	0.00202	0.09898
P01009	-3.030096	556.3295	0.00256	0.10752
P02675	-2.840552	457.5436	0.00470	0.172725
P05090	-2.746976	664.8847	0.00618	0.184044
P25311	-2.742384	659.7582	0.00626	0.184044
Q6ZS72	-2.601179	668.7169	0.00950	0.23618
P02741	2.596366	624.1705	0.00964	0.23618
P01023	-2.530822	651.5998	0.0116	0.2604
P06396	-2.507754	665.6218	0.0124	0.2604
P14543	2.441361	665.9270	0.0149	0.29204
P02679	-2.347287	429.7639	0.0194	0.3342316
P02748	-2.316086	671.9688	0.0209	0.3342316
P02776	-2.312238	632.6814	0.0211	0.3342316
Q86VB7	2.302403	660.6239	0.0216	0.3342316
P04114	-2.226078	659.2533	0.0263	0.38661
Q9UGM5	2.149521	670.5964	0.0320	0.410375
P02750	-2.146205	662.0062	0.0322	0.410375
Q9HDC9	-2.131135	664.1009	0.0334	0.410375
P27169	-2.130542	671.3090	0.0335	0.410375
Q15113	-2.089336	668.6740	0.0371	0.4206462
P01008	2.087374	671.8398	0.0372	0.4206462
Q9Y6U3	-2.062030	667.7270	0.0396	0.4312
Q12805	-2.041367	665.2313	0.0416	0.43512
Q13740	2.019846	670.1554	0.0438	0.43512
P00742	-2.014102	671.8587	0.0444	0.43512
P63267	-1.981180	579.9825	0.0480	0.4461882

Table S2. Feature-importance rankings for the best-performing Random Forest, Gradient Boosting, and Neural Network classifiers.

Rank	RF-4	GB-4	NN-4
1	ApoE4	ApoE4	P04114
2	P01024	P02741	P01024
3	P02741	P04004	P08603
4	P08603	Q13740	P01008
5	Q13740	P08603	P01042
6	P04004	Q6ZS72	P05090
7	Q6ZS72	P01024	P04004
8	P02748	P01009	Q15113
9	P01009	P02750	Q13740
10	P00742	P01008	Q12805
11	Q9HDC9	P01023	P02750
12	P05090	P63267	P06396
13	P27169	Q9HDC9	P01009
14	P63267	P27169	P02679
15	P01023	Q12805	P14543
16	Q15113	P05090	P02671
17	P01008	P02748	ApoE4

Rank	RF-4	GB-4	NN-4
18	Q9UGM5	P02675	P01023
19	Q86VB7	P04114	Q9Y6U3
20	Q12805	P25311	P02675
21	P02750	P01042	P02741
22	P25311	Q9UGM5	P02776
23	P01042	P00742	Q9HDC9
24	P14543	Q86VB7	Q86VB7
25	P02671	Q15113	P63267
26	Q9Y6U3	P02671	P25311
27	P06396	P02679	Q6ZS72
28	P04114	P14543	P27169
29	P02776	P02776	Q9UGM5
30	P02679	Q9Y6U3	P00742
31	P02675	P06396	P02748

Table S3. Stratified bootstrap feature-stability analysis of top-ranked proteins across resampled Random Forest and Gradient Boosting model-training iterations.

Bootstrap stability analysis used 1000 stratified resamples. Frequencies indicate the percentage of bootstrap iterations in which each protein appeared among the top 10 ranked features. Combined RF/GB frequency represents the pooled selection frequency across 2000 RF/GB model-iterations and is shown with Wilson 95% confidence intervals. Median rank and interquartile range were calculated across iterations in which the protein was selected among the top 10 features. The top-10 threshold was defined before the stability analysis and was not selected using the bootstrap results.

Feature	Gene label	In final signature	FDR significant original screen	RF top-10 frequency (%)	GB top-10 frequency (%)	Combined RF/GB top-10 frequency, % (95% CI)	Median rank when selected	IQR rank when selected	Interpretation
ApoE4	APOE4	Yes	Yes	100.0	100.0	100.0 (99.8-100.0)	1	1-1	Highly stable
P01024	C3	Yes	Yes	82.8	65.1	74.0 (72.0-75.8)	3	2-6	Moderately stable
P02741	CRP	Yes	No	56.5	41.5	49.0 (46.8-51.2)	5	3-7	Variable but recurrent
P08603	CFH	Yes	Yes	51.5	43.6	47.5 (45.4-49.7)	5	4-8	Variable but recurrent
P01009	SERPINA1	Yes	No	34.5	33.7	34.1 (32.1-36.2)	6	4-8	Not stable
P04004	VTN	Yes	Yes	38.0	19.0	28.5 (26.6-30.5)	6	4-8	Not stable
Q13740	PON1	Yes	No	17.6	12.1	14.8 (13.4-16.5)	6	4-9	Not stable
Q6ZS72	C1QTNF5	Yes	No	9.4	9.8	9.6 (8.4-11.0)	7	5-9	Not stable
P02671	FGA	No	Yes	25.8	15.5	20.6 (18.9-22.5)	6	4-9	Not stable

Abbreviations: CI, confidence interval; FDR, false discovery rate; GB, Gradient Boosting; RF, Random Forest. Interpretative labels were based on combined RF/GB top-10 frequency: highly stable >=80%, moderately stable 60-79.9%, variable but recurrent 40-59.9%, and not stable <40%.
